# Modeling biochemical transformation processes and information processing with Narrator

**DOI:** 10.1186/1471-2105-8-103

**Published:** 2007-03-27

**Authors:** Johannes J Mandel, Hendrik Fuß, Niall M Palfreyman, Werner Dubitzky

**Affiliations:** 1 Dept. of Biotechnology & Bioinformatics, Weihenstephan University of Applied Sciences, 85350 Freising, Germany; 2 School of Biomedical Sciences, University of Ulster, Coleraine BT52 1SA, Northern Ireland

## Abstract

**Background:**

Software tools that model and simulate the dynamics of biological processes and systems are becoming increasingly important. Some of these tools offer sophisticated graphical user interfaces (GUIs), which greatly enhance their acceptance by users. Such GUIs are based on symbolic or graphical notations used to describe, interact and communicate the developed models. Typically, these graphical notations are geared towards conventional biochemical pathway diagrams. They permit the user to represent the transport and transformation of chemical species and to define inhibitory and stimulatory dependencies. A critical weakness of existing tools is their lack of supporting an integrative representation of transport, transformation as well as biological information processing.

**Results:**

Narrator is a software tool facilitating the development and simulation of biological systems as *Co-dependence models*. The *Co-dependence Methodology *complements the representation of species transport and transformation together with an explicit mechanism to express biological information processing. Thus, Co-dependence models explicitly capture, for instance, signal processing structures and the influence of exogenous factors or events affecting certain parts of a biological system or process. This combined set of features provides the system biologist with a powerful tool to describe and explore the dynamics of life phenomena. Narrator's GUI is based on an expressive graphical notation which forms an integral part of the Co-dependence Methodology. Behind the user-friendly GUI, Narrator hides a flexible feature which makes it relatively easy to map models defined via the graphical notation to mathematical formalisms and languages such as ordinary differential equations, the Systems Biology Markup Language or Gillespie's direct method. This powerful feature facilitates reuse, interoperability and conceptual model development.

**Conclusion:**

Narrator is a flexible and intuitive systems biology tool. It is specifically intended for users aiming to construct and simulate dynamic models of biology without recourse to extensive mathematical detail. Its design facilitates mappings to different formal languages and frameworks. The combined set of features makes Narrator unique among tools of its kind. Narrator is implemented as Java software program and available as open-source from .

## Background

### Motivation

One goal of investigating the dynamic behavior of biochemical systems and processes is to understand biological causality in terms of regulation and control mechanisms. Computerized mathematical models of such dynamic behavior have become an important methodology in the pursuit of this goal. Many modeling and simulation tools have been reported in the literature. The Systems Biology Markup Language (SBML) Web site [1] lists more than 100 such tools, all of which adhere to the SBML standard, facilitating the exchange of biochemical reaction network models. In addition to systems modeling and simulation tools, the SBML site posts a large number of tools addressing other tasks, including tools to convert SBML files into other languages or formats (such as R [2], Matlab [3] or Mathematica [4]), to modify and combine different SBML files (see for example SBMLmerge [5]), and to interactively analyze and explore a large number of already developed biological models (e.g., Web-based kinetic modeling [6,7] and kinetic model databases or modelbases [8]).

Several excellent tools have been developed for modeling and simulating biochemical networks, some of which provide sophisticated, GUI-based design tools for visual model development. Most of these tools are freely available as software packages or as online tools. Examples include Copasi [34], Virtual Cell [9], JDesigner [10], CellWare [12] and CellDesigner [11,33], which are well developed and widely used in the systems biology community.

A critical weakness of the symbolic notations of these tools is their lack of supporting an integrative representation of biological transformation and transportation processes as well as biological information processing.

Within the context of this work, we define *species transformation *as the alteration or change of species participating in biochemical processes. Examples of species transformation include stoichiometric reactions, enzymatic reactions, synthesis and degradation processes or modification processes such as methylation and phosphorylation. *Biological transport *refers to processes that transport organic, biochemical or inorganic substances between or within cells. Protein translocation across subcellular compartments or material flow through biological membranes are examples of biological transport. *Biological information processing *controls the processes involved in species transformation and species transport. By processing the information provided by one or more information sources and by regulating biological transport and transformation processes accordingly, biological systems can control both the maintenance of a state and also state transitions from one state to the next. Based on this definition information sources can be of different biological and non-biological nature such as a set of transcription factors controlling the expression of a gene, or physical parameters influencing a biological process (e.g. temperature, osmotic pressure or pH-values). Typical examples of biological information processing include gene regulatory processes and cell-cell or cell-environment interactions mediated through receptor proteins. To simultaneously model species transformation and biological information processing is important because only this integrative approach can describe biochemical processes including the influencing factors that control such processes and their interplay. For example, a model of osmotic shock in yeast should capture species transformation and information processing because it is not possible to formulate the causal dependencies of osmotic shrinking and swelling of yeast cells by defining transformation processes exclusively. Instead the processing of internal and external ion concentration levels is fundamental in the response processes initiated by the yeast cells and thus must be integrated into the model to provide contextual causality of the underlying system. While the main focus of most existing tools is placed on the description of species transformation, the modeling of information processing which controls such transformations is an important feature missing in those tools. The main novelty of the Narrator tool is that it facilitates the integrative modeling and simulation of biological transformation, transport and information processing.

**Figure 1:**
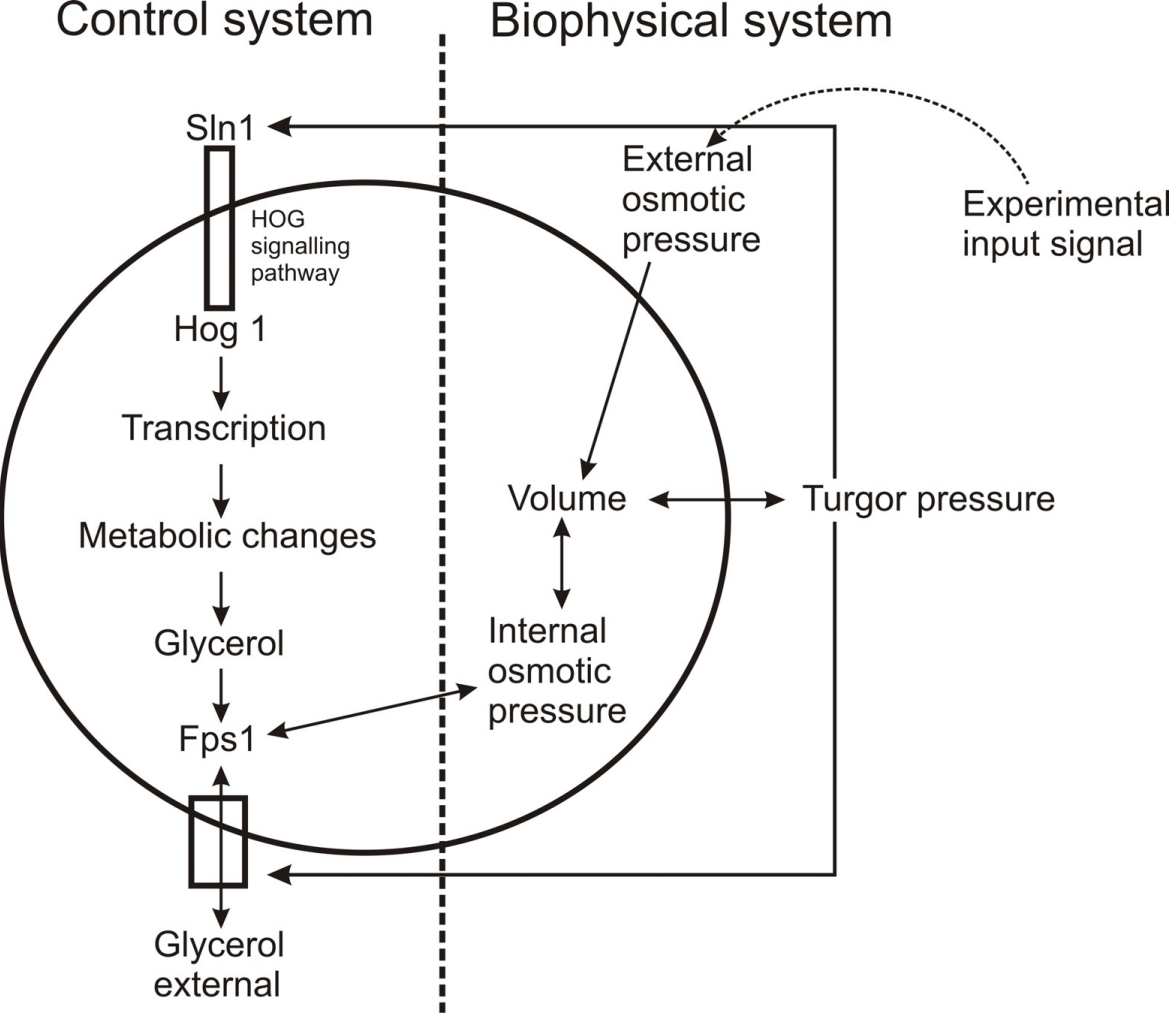
**Overview of osmoregulation in yeast (based on Figure 1 in [44])**. The model depicted in the diagram shows the closed loop from the osmosensor at the membrane, via signal transduction, gene regulation of metabolism and the feedback effect of internal glycerol levels on the osmotic pressure and cellular volume [43]. The model illustrates the coupling of a biochemical (control) with a biophysical system.

The following illustrative example is to demonstrate the necessity of an integrative modeling approach that combines transformation and information processing. It is based on a model of osmotic shock in yeast and was developed by Klipp and coworkers [15]. This model, illustrated in Figure 1, integrates biochemistry (species transformation, gene regulation, metabolism) and biophysics (volume and internal and external osmotic pressure). While the control system involving signal transduction, gene regulation, metabolic transformation and glycerol transport, can be seamlessly described with the abovementioned tools, it is difficult to represent the biophysical system, which involves information or signal processing, with the graphical notations provided by these tools. The development of the Narrator software tool was driven by the need to model and simulate such multi-modal biological systems.

### Co-dependence models

The Narrator tool presented in this paper implements the *Co-dependence Methodology *[14], which consists of a set of concepts and tools facilitating the modeling and simulation of biological systems and processes. Among others, the development of this methodology was guided by the following needs: The methodology should

1. Enable life scientists to express the full complexity of biological functioning, including species transport, transformation and information processing which guides these processes.

2. Provide a visual or graphical notation suitable for use in informal pen-and-paper discussions and implementation as sophisticated graphical user interface of computerized modeling tools.

3. Be executable on a computer to enable the testing of proposed dynamics *in silico*.

**Figure 2:**
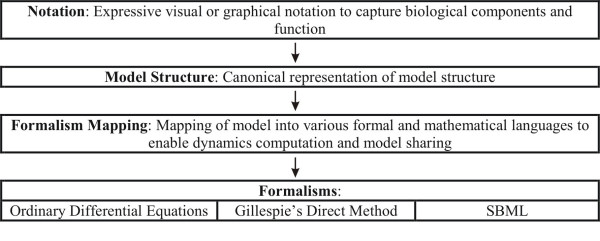
**The Co-dependence Methodology**. Depiction of Co-dependence Methodology's conceptual layers and components: notation, model structure, formalism mapping and formalisms.

The conceptual architecture of the Co-dependence Methodology can be divided into four layers (Figure 2). Narrator's Notation layer at the top provides a flexible and intuitive visual or graphical notation facilitating the specification of a wide range of biological functioning and information processing mechanisms. Each model in Narrator is captured at the canonical Model Structure layer, which represents the basic modeling abstractions and concepts (e.g. species, process). The components at the Model Structure level are also used to realize the mapping (depicted in the conceptual structure by the Formalism Mapping element) of the model into various formal or mathematical frameworks, schemes or languages. Finally, the Formalism layer represents the different types of formal and mathematical languages in which models developed in Narrator can be expressed to facilitate computation and sharing. Currently three of such formalisms are supported: ordinary differential equations, Gillespie's Direct Method and SBML.

### Co-dependence Notation

Most notations currently used in systems biology tools facilitate the description of conventional biochemical pathways and are based on mathematical *bipartite graphs *or *directed hypergraphs*. A bipartite graph (or bigraph) is defined by a set of graph vertices (nodes) decomposed into two disjoint sets such that no two graph vertices within the same set are adjacent, i.e., they are not joined by a graph edge (link or arc). A directed hypergraph is a graph in which generalized edges (also known as hyperarcs) may connect more than two nodes. The two types of graphs have been shown to be equivalent when used for representing transport and transformation of chemical species [13,40]. In bipartite graphs, there are two kinds of node, and no edge can relate nodes of the same kind. This is useful for linking biological species to processes or reactants to products. Hyperarcs of directed hypergraphs can be used to relate *sets *of reactants to *sets *of products, where each set belong to the same kind of node. Additionally, both notations can represent inhibitory and stimulatory dependencies between chemical species and their transformation processes. Petri nets [18], the E-Cell process variable model or graphical notations used in JDesigner or CellWare belong to this class of graph.

**Figure 3:**
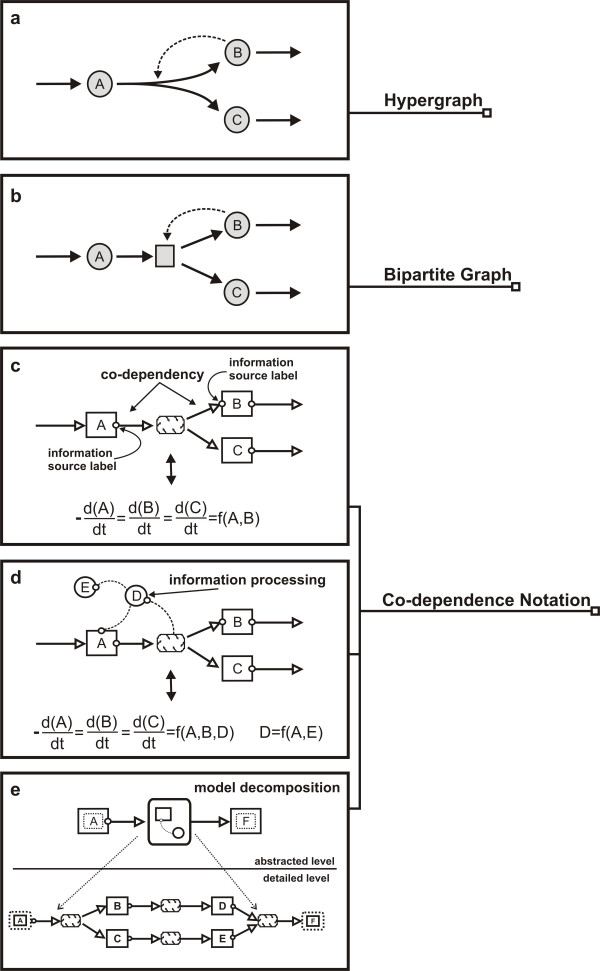
**Novel aspects in the Co-dependence Notation**. The diagram shows a simple model involving three biochemical species A, B and C rendered as a directed hypergraph, a bipartite graph and rendered in the Co-dependence Notation. Further the mapping of the Co-dependence Notation into the ordinary differential equation (ODE) formalism is depicted. Similar to bipartite graphs, the Co-dependence Notation comprises two different kinds of node, called species and process. Rectangles (Co-dependence Notation part at the bottom of the diagram, c and d) represent molecular species, clouds denote transformation processes, thick arrows represent material flow and thin dashed lines represent informational flow. One novel aspect in the Co-dependence Notation is the use of small, unfilled circles to label information sources i.e. to label entities that play a functional role in their associated process. Consequently a thick arc in conjunction with a small circle represents a co-dependency between the species and process nodes associated by the link. In this co-dependent relationship, the species influences as information source the process that is transforming the species. As indicated in the diagram sections c and d, the visual emphasis of information sources enables the mapping of the Co-dependence Notation into ODEs. A further novel aspect in the Co-dependence Notation is the optional use of information processing nodes to represent constants or computed logical entities such as rate constants, external stimuli or genetic switches. Information processing nodes also belong to the class of process nodes yet are rendered as large circles. Thus, links in Co-dependence models cannot only connect species nodes with process nodes but can also directly relate two or more process nodes with each other. This facilitates the representation of both species transformation and information processing within a single conceptual framework. The diagram section E shows how Co-dependence models decompose complex systems into simpler elements called *compartments*.

As illustrated in Figure 3, Co-dependence Methodology models comprise, similar to bipartite graphs, two different kinds of node, called species and process. In their notational rendering, rectangles represent molecular species and clouds denote transformation or transport processes. Directed links are used to describe a dependency between two nodes and represent either material or informational flow. Thick-lined arrows represent material flow and are always connected between reaction and species nodes. Thin-lined, dashed links represent informational flow and can connect species with process nodes or directly relate two process nodes.

A novel aspect in the Co-dependence Notation is the explicit labeling of *information sources *using small, unfilled circles. Interpreted dynamically, information sources are considered as mathematical variables that are integrated into the function or rate law of their associated process. This is illustrated in Figure 3c by the small Co-dependence model and its corresponding equations. Species A and species B, for instance, have an influence on the process transforming species A, B and C.

A thick arc in combination with a small circle indicates that there exists material as well as informational flow between a species and a process node. In this *co-dependent *relationship between a species and a process, the species influences the process that is transforming the species.

Co-dependence models are fully isomorph to systems of ordinary differential equations (ODEs). The rate of change of each species represents an ODE and, as shown in Figure 3c and Figure 3d, it is the visual emphasis of information sources in the Co-dependence Notation (see information source labels) that enables the presentation of the individual factors playing a functional role in the ODEs. Process nodes that are representing the transformation of species are then describing the coupling of the ODEs.

An additional novel aspect of the Co-dependence Notation is the optional use of *information processing nodes *to represent constants or computed logical entities such as rate constants, external stimuli or genetic switches. Information processing nodes also belong to the class of process nodes, yet, as shown in Figure 3d, they are rendered as large circles. Information processing nodes also serve as information sources and can be linked to other process nodes accordingly. Thus, links in Co-dependence models cannot only connect species nodes with process nodes but can also directly relate two or more process nodes with each other. This facilitates the representation of both species transformation and information processing within a single conceptual framework.

From a graph-theoretical point of view the visual representation of information processing is made possible by defining a link or edge type which is able to connect process and species nodes as well as relating two process nodes. Such an informational relation between two process nodes has no direct biochemical interpretation and therefore is not considered in notations derived from biochemical pathway diagrams. Yet, as we shall demonstrate, this extension is extremely useful when incorporating abstractions or additional information into models as is for example required when integrating biochemical and biophysical aspects in one model. Consequently, in Co-dependence models, process nodes describe both the transformation of concentration levels of their participating species as well as the processing and supply of information. In particular, the ability to link processes *informationally *is of considerable value in scenarios illustrated by the following four scenarios.

#### Scenario 1: Incorporating exogenous variables

**Figure 4:**
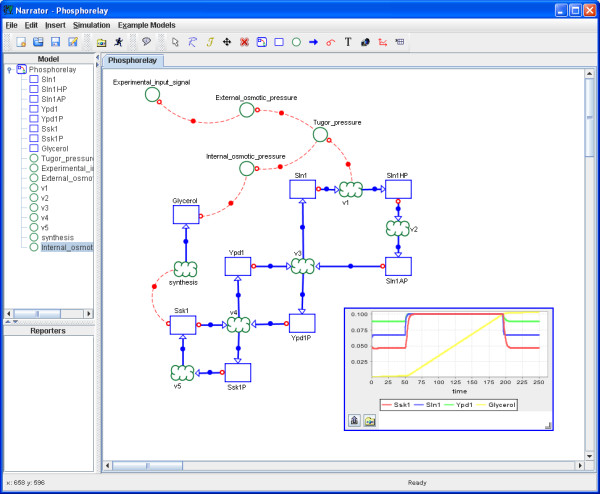
**Describing the phosphorelay mechanism of the HOG signaling pathway taken from [15]**. The shown Co-dependence model describes graphically the individual species and their state transitions of the phosphorelay mechanism of the yeast high osmolarity glycerol (HOG) pathway. It further describes that species *SLN1 *is directly affected by the concentration difference between ions in the extracellular and intracellular environment. The dependency of the process node *v1 *is described in detail with a sequence of processing steps involving the nodes *osmoticShock*, *extracellularConcentration *and *osmolarity*.

A link between two process nodes can describe how exogenous factors affect a biological system. For example, Klipp et al. describe the phosphorelay mechanism of the yeast high osmolarity glycerol (HOG) pathway which controls the transcriptional response of yeast cells to high osmolarity [15]. In the phosphorelay model depicted in Figure 4, we present graphically the individual species and their state transitions of the phosphorelay mechanism. We further show that species *SLN1 *is directly affected by the *Turgor pressure *which again depends on the *Internal *and *External osmotic pressure*. Thus, we can visually describe which species is contributing as an osmosensor to the regulation of the HOG pathway and how this species depends on various biophysical influences.

#### Scenario 2: Describing detailed steps of information processing

Figure 4 illustrates what we understand by the term *detailed steps of information processing*. Sometimes it is convenient to elaborate a single informational dependency into a sequence of processing steps. For example, in Figure 4, where the simple dependency of process *v1 *on the species *glycerol *has been expanded into the more detailed description involving *Experimental input signal*, *External *and *Internal osmotic pressure*, and *Turgor pressure*. These detailed steps of information processing allow us to visually close the loop between the biological system *internal osmotic pressure *and its environments (*external osmotic pressure*).

#### Scenario 3: Reducing diagram complexity with auxiliary processes

In ODE-based modeling, *differential-algebraic equations *are often used to reduce the complexity of the differential equations by simplifying their right-hand expressions with auxiliary variables. Biochemical models frequently make use of this method. However, although SBML's AssignmentRule tag provides support for it, the process nodes in Narrator can improve the readability of equation systems by structuring information processing in a similar way. Differential-algebraic equations thus translate to Co-dependence models without any increase in diagram complexity.

**Figure 5:**
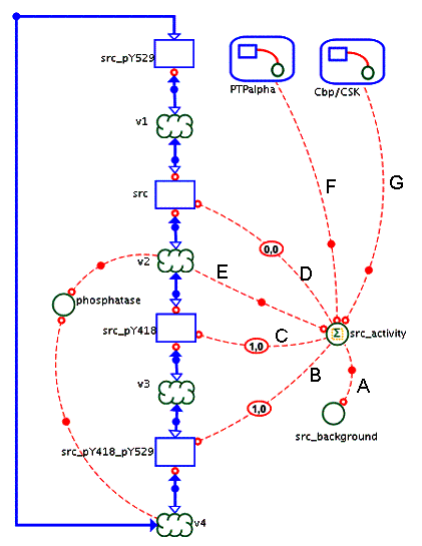
**Describing the mitotic activation of the tyrosine kinase Src (excerpt from [16])**. The process node *src_activity *is used to calculate the total enzymatic activity of the kinase Src, which is represented in four different phosphorylation states. The value of the *src_activity *node is then reused in processes that represent reactions catalyzed by this kinase, such as autophosphorylation (E), phosphorylation of PTPα (F) and Cbp (G). This graphical notation directly corresponds to the common method of modeling biochemical systems using differential-algebraic equations.

In signaling models, auxiliary variables are sometimes used to describe the concentration, activity or any other property of a protein which is represented as multiple species due to the existence of protein modifications. For example, in a model describing the mitotic activation of the tyrosine kinase Src [16], a process node called *src_activity *is used to calculate the total enzymatic activity of that kinase. In the model depicted in Figure 5, Src is represented in four different phosphorylation states. Due to allosteric effects each state contributes to kinase activity to a different degree (represented by different weights of informational links A-D). The value of the *src_activity *node is then reused in processes that represent reactions catalyzed by this kinase (links labeled E, F, G in Figure 5).

**Figure 6:**
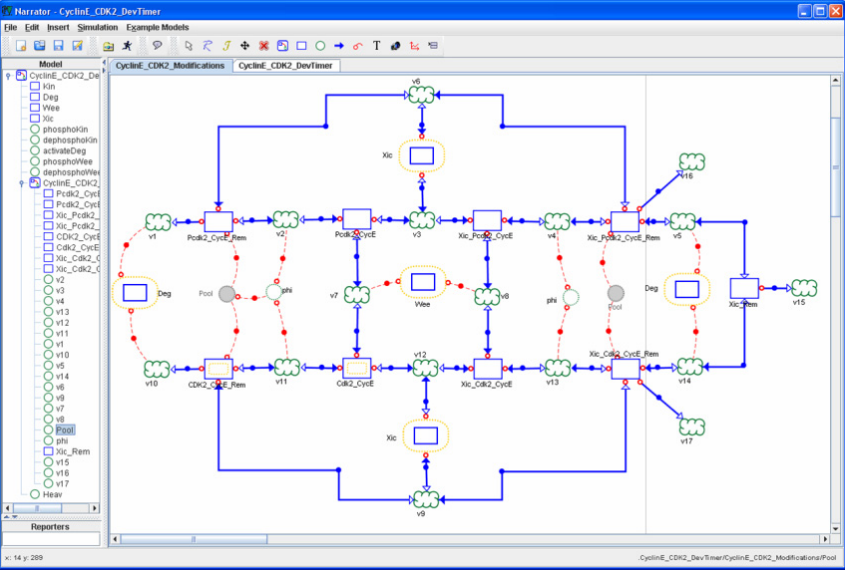
**Describing the cycle model of Xenopus embryos taken from [17]**. Different states and state transitions of CyclinE:Cdk2 dimers taken from the Cycle model of Xenopus embryos [17]. Using the auxiliary process Pool (grey shaded and replicated) to structure the use of all removed forms of Cyclin:Cdk2 dimers.

Similarly, in the Co-dependence interpretation of the cell cycle model describing chemical oscillations in the early embryonic development of the frog *Xenopus laevis *embryos [17] (shown in Figure 6), we also make use of an auxiliary process node to structure the composition and usage of the *Pool *of all removed forms of *Cyclin:Cdk2 *dimers contained in the model. This variable *Pool *is defined as auxiliary variable in the differential-algebraic equation system [17] and could be seamlessly translated into the Co-dependence Notation.

Both examples (Figure 5 and Figure 6) illustrate how the proliferation of links can be avoided by introducing auxiliary process nodes.

#### Scenario 4: Emphasizing important process parameters

Quantitative models of biological processes strongly depend on constant parameters such as Hill coefficients, half-saturation constants, rate constants or specific growth and degradation rates. In a Co-dependence model we can describe selected parameters explicitly with process nodes to emphasize their importance to the overall model or to allow direct manipulation of the parameter value via our software tool Narrator.

In all abovementioned examples it is up to the modeler to decide which level of detail is appropriate for a model. For example, if we incorporated only biochemical transformation processes into our model it would look very similar to other bipartite approaches such as Hybrid Object Net [18] or E-Cell [19].

On the other hand, information processing structures resemble the models of the *System Dynamics *methodology. In this method, developed by Forrester [32], dynamic system models are built with stock and flow diagrams which implement the *principle of accumulation *[20]. Here, dynamic behavior is described by flows that accumulate in stocks and a third node type called *converter *can be used to describe graphically the information processing in a system. This graphical notation however is not suitable for modeling stoichiometric relations in metabolic reactions and thus is not applicable to biochemical modeling [21].

As shown in Figure 3e, the Co-dependence Methodology provides a mechanism for decomposing complex systems into simpler elements called *compartments*. Compartments may themselves contain other nested elements [14]. This hierarchical structure allows to model systems of any appreciable complexity and is also implemented in Narrator. This mechanism can be used to encapsulate parts of a model that correspond to one cell compartment but it can be also useful to provide different levels of detail of a model or to integrate a set of models as submodels into one model.

## Implementation

**Figure 7:**
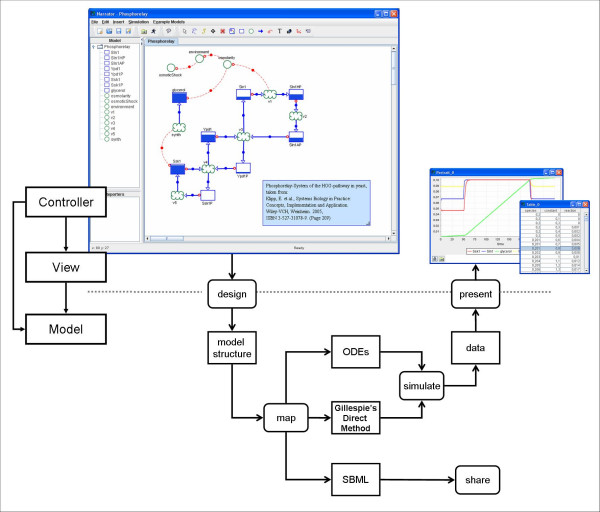
**Typical Narrator workflow**. The graphical user interface of the design tool facilitates the interactive construction and manipulation of models based on the Co-dependence Notation. The models designed with this tool conform to the syntax and semantics of the notation. Narrator can map models designed in this way to different underlying formal languages and mathematical schemes such as ODEs and simulate the models' dynamics using numerical integration. The generated data can be presented for analysis and evaluation.

Narrator primarily implements the Co-dependence Methodology which comprises the four components depicted in Figure 2: graphical notation, model structure, formalism mapping and, so far, three implemented mappings (ODE, Gillespie, SBML). The visual or graphical notation defines the symbols and diagrammatic elements and the syntactic and semantic rules on how to use these to compose visual depictions of Co-dependence models. The model structure components describe the data and information structures facilitating the instantiation of graphically specified Co-dependence models and their mapping into a mathematical formalism or representation language, providing computability (simulation) and shareability (information exchange) of Co-dependence models. Currently three mappings have been implemented. Details of the Co-dependence Methodology have been reported previously in the literature [14]. The software architecture of Narrator is depicted in Figure 7. In the diagram the four main conceptual elements of the Co-dependence Methodology are clearly visible.

### Architecture and Workflow

Narrator is implemented in Java 5 on the basis of the model-view-controller design pattern, which separates core (application) model functionality from the presentation and control logic that uses this functionality [45].

Figure 7 depicts a typical Narrator workflow. Via the user interface, the modeler designs and manipulates dynamic models according to the Co-dependence Methodology [14]. This involves defining the model structure and assigning initial concentration values and functions to all species and process nodes. Narrator provides a click-drag-drop environment for placing and controlling nodes and links. Narrator contains its own interpreter system which compiles functions from a mathematical expression in string form into an execution tree. The instructions of such trees are executed during simulation to return the runtime function values. A set of classes for different mathematical operators has been created using JavaCC [22] technology. To facilitate simulation, the software tool implements a set of numerical integration methods. Currently these methods include Euler's method, and 2^nd ^and 4^th ^order Runge-Kutta and Runge-Kutta-Fehlberg with adaptive step size (for a detailed description of the integration methods and their implementation see for example [46]). Additionally, Narrator implements Gillespie's direct method to simulate stochastic processes [23]. To examine the resulting dynamics of a model the user can create time-series diagrams, phase portraits [47] and tabular output.

### Narrator models

**Figure 8:**
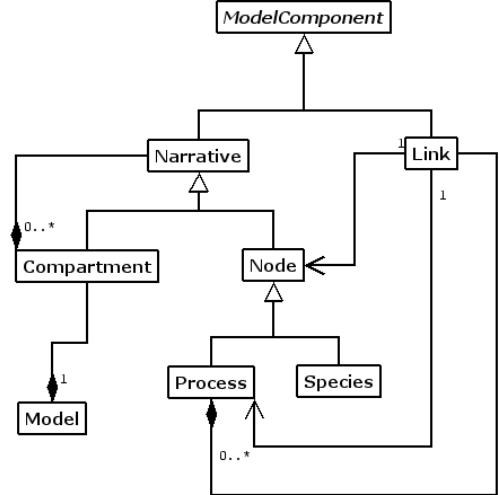
Class diagram describing the structure of a Narrator model.

Figure 8 depicts the structure of Java classes that implement the concepts needed to represent Narrator models. The classes Species, Process, Compartment and Link reflect the Co-dependence Methodology's main conceptual model components *species*, *process*, *compartment *and *link*. Compartment objects encapsulate a finite number of Species and Process objects and can be used to model cellular compartments or to decompose a model into constituent elements. Link objects connect Process objects with Species or other Process objects. They model the propagation of change between species and processes (e.g. biochemical transformation) or the transport of information to the processes (e.g. stimulation, inhibition or information processing).

Each model created using Narrator contains one root compartment, which in turn can contain additional compartments, processes and species. The relationship among the classes Link, Process and Species further enables the mapping to and execution of numerical simulations of Narrator models directly within the model structure. Process objects coordinate via Link objects the augmentation and depletion of the concentration levels represented by Species objects. As shown in Figure 8, Link objects can associate Node and Process objects for this purpose.

Similarly, the class structure can be used to instantiate and simulate stochastic processes. Currently, Narrator implements Gillespie's direct method [23] where the runtime function values in the process nodes determine the probability of each reaction to occur in a certain time interval. This time interval is also calculated probabilistically, and when a reaction occurs, all participating Species objects are updated according to their stoichiometry, which can be defined through Link objects.

### Reporters

Narrator provides two reporting components to present the resulting dynamics data of a Narrator model. These are portraits and tables respectively. Portraits are based on the open-source chart library jfreechart [24] and can either be used to generate time-series plots or phase plots. Tables present the time-dependent development of selected variables and can export their information as ASCII-formatted files.

### Animation

**Figure 9:**
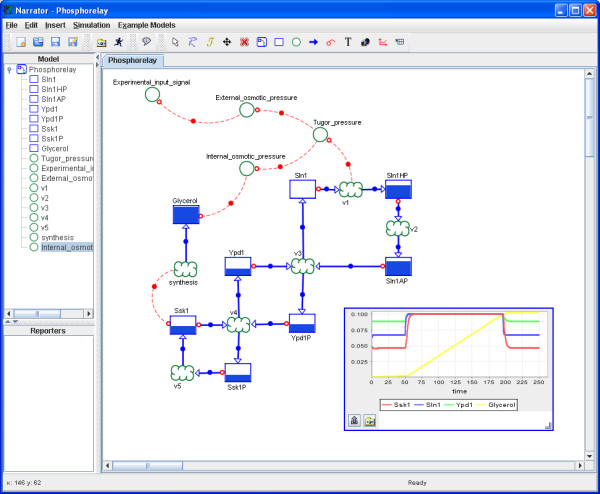
**Animation of species nodes**. Phosphorelay mechanism of the HOG signaling pathway taken from [15] with species animation. Filled species nodes indicate that they have reached their maximal concentration level relatively to their concentration development within the simulation.

The user can visually simulate the dynamics of the model by animating the species nodes of the graphical network. As shown in Figure 9, species nodes are graphically filled and depleted during simulation and thus facilitate visual monitoring the overall dynamics of the system.

### SBML Export and Import

To facilitate a standard-compliant exchange of Co-dependence models, Narrator provides an export and import mechanism for storing Co-dependence models in SBML level 2 version 1 [48]. However, Narrator does only embrace elements of the SBML language that match to elements of the Co-dependence Methodology. Essentially, these are species, reactions, parameters, rules and the assignment of initial values or functions for these components. Narrator does currently not support unit and function definitions and provides no automatic mechanisms to layout imported SBML models. Consequently, Narrator cannot read all SBML models created by other SBML-platforms, but SBML models created with Narrator are SBML compatible.

For transforming mathematical String expressions into MathML we make use of the open-source library jeks [25] and for reading MathML elements we us the class jigcell.sbml2.math.MathMLConvertor which is part of the open-source distribution JigCell [35].

## Results

To evaluate Narrator, we described and simulated a model of the protein kinase C (PKC) pathway taken from Pettinen et al. [26] which used this pathway model as a test case for comparing and evaluating different simulation tools for biochemical networks. In their study Pettinen et al. emphasize the importance of usability and the benefits of exchanging standards, and conclude that simulation tools should support the integration of exogenous variables for the modeling of stimuli since biological systems are not separated from their environment. Another important feature discussed in this article is the automatic estimation of parameters where no information is available.

**Figure 10:**
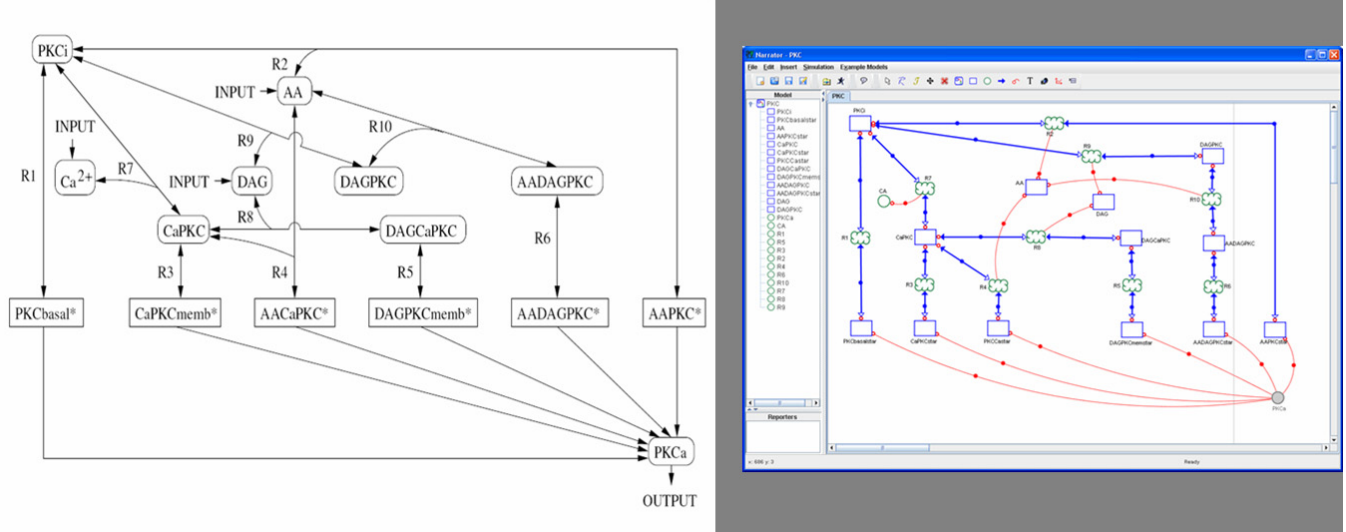
**Protein kinase C pathway taken from [27]**. Left: Model of the protein kinase C pathway taken from [27]. This model was used in [26] as a test case for validating different simulation tools for biochemical networks. Right: PKC model described with Narrator.

**Figure 11:**
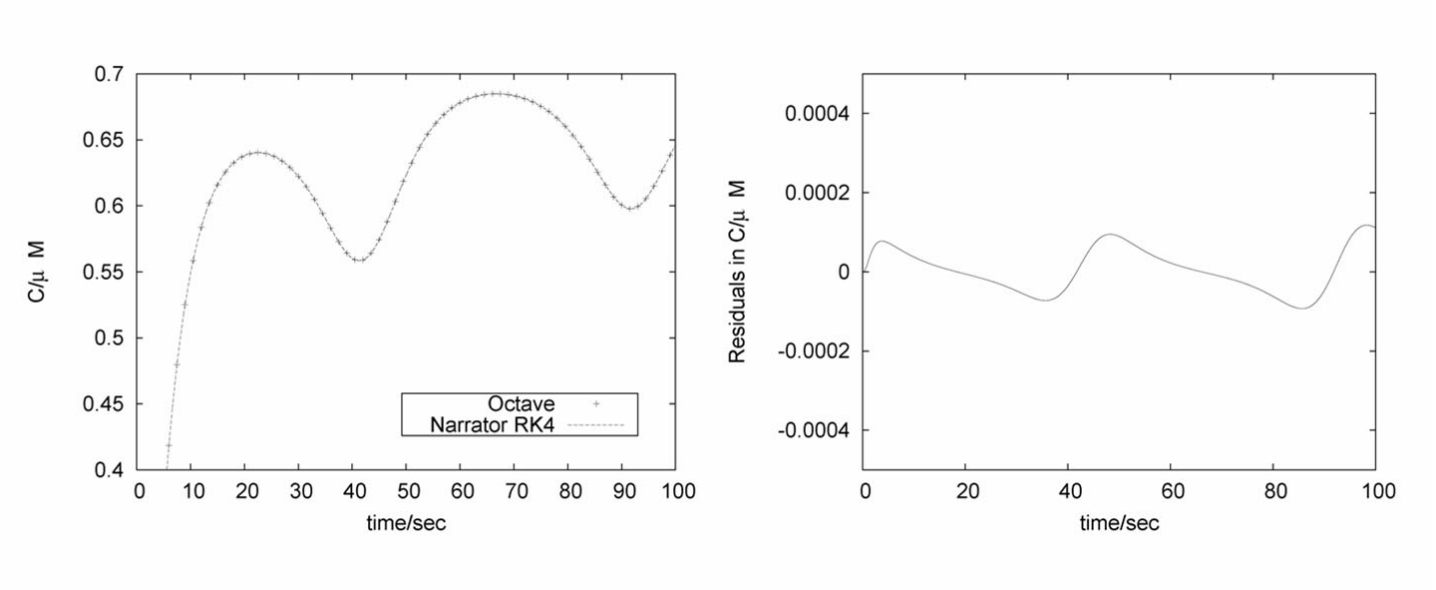
**Validating Narrator's Runge-Kutta 4 implementation**. Simulation concentrations of active PKC using Octave and Narrator (left). The residual plot (right) shows minimal differences between the simulation results and may be due to the different numerical integration methods.

The PKC model contains 11 differential equations and two further reactants which are kept constant in the simulation. Based on the ODE definition of the model [26] we built the PKC model with Narrator (see Figure 10) and simulated its dynamics. To evaluate the integration methods of Narrator, we compared the simulation results of Narrator using Runge-Kutta 4 and the numerical computation tool Octave [28] which uses the Livermore Solver for Ordinary Differential Equations [29]. As Figure 11 shows, minimal differences between the two simulation runs could be observed. For running this model, the simulation time of Octave equals the simulation time of Narrator when using the implemented Runge-Kutta 4 method and step size of 0.005 seconds. Simulation time using Copasi however, showed to be significantly faster.

**Figure 12:**
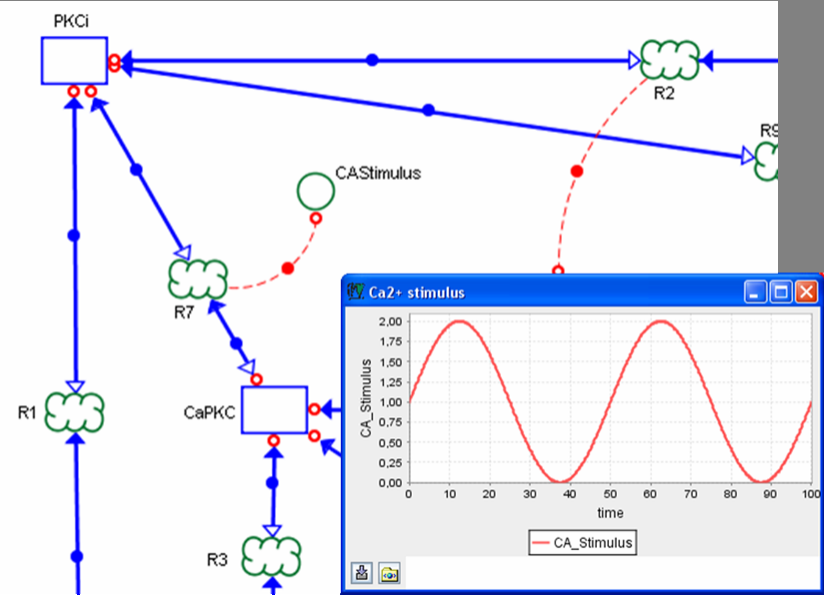
**Modeling stimuli with Narrator**. Ca2+ stimulus for the PKC pathway described with the process CAStimulus linked to the reaction R7.

A larger section of the PKC model described with Narrator is shown in Figure 12, where we use the process *CAStimulus *linked to the reaction *R7 *to model the exogenous Ca^2+ ^stimulus for the PKC pathway following a sine wave with time period of 50 seconds.

We used the same model to evaluate the SBML compatibility of Narrator. The PKC Narrator model was exported into SBML and the generated XML file was validated with the SBML Validator available on the SBML Web site [30]. SBML confirmed that the exported file adhered with the SBML format. We also imported this XML file successfully into the SBML platform Copasi after setting the stimulus of CA^2+ ^to a constant value.

In another recent study by Alves and co-workers [42], 12 kinetic modeling tools of biochemical networks have been compared and evaluated according to a variety of criteria addressing systems requirements, cross-compatibility, diagrammatic user interfaces or analytical capabilities of the simulators. As shown in Table 1, we have used these criteria to summarize the functions and features of Narrator.

**Table 1:** Feature and requirement summary of Narrator. Summary of Narrator features and requirements according to criteria used in [42], addressing systems requirements, cross-compatibility, diagrammatic user interfaces or analytical capabilities of the simulators.

**Type/Name of Criterion**	**Value of Criterion**
Website	Yes ()
Hardware requirements	~10 MB disk space
Input (model definition)	Diagrammatic
Software requirements	JRE version 1.5
Operating System	OS independent
Import	SBML
Export	SBML
Open source	Yes (Sourceforge)
Systems Biology Workbench	No
Graphical representation of multiple compartments	Yes
Representation of interface reactions	Yes
Representation of multiple reactant types	No
Representation of regulatory interactions	Yes
Automated network layout	No
Library of predefined rate expressions	No
Editable list of all parameters/initial conditions	No
Implementation of non-integer stoichiometries	Yes
Ordinary differential equations	Yes
Partial differential equations	No
Stochastic	Yes
Automated set-up of compartmentalization	No
Direct calculations of steady states	No
Steady-state stability analysis	No
Steady-state sensitivity analysis	No
Time-dependent sensitivity analysis	No
Automated parameter scanning	No
Time-dependent perturbations	Yes
Parameter estimation/optimization	No

Narrator provides a set of example models which are directly accessible via the menu bar. Some models are taken from Klipp et al. [15] and demonstrate Narrator's ability to model and exchange small systems and to simulate their dynamic behavior. The other models are example models developed for this study and in earlier work introducing the Co-dependence Methodology [14].

## Discussion

In general, graphical notations of systems biology tools are designed to capture critical elements that determine the *structure *and govern the *behavior *of a biological system or process. Some of the existing notations and tools emphasize the structural and others the behavioral dimension. Typically, structure-oriented notations use a plethora of graphical symbols and notational conventions to characterize qualitative details of species types and their biochemical transformations. Dynamics-oriented notations, on the other hand, capture information relevant mainly to the dynamic aspects of a system and usually subsume different species and transformation types within a small collection of graphical symbols (see for example bipartite graphs and hypergraphs in Figure 3ab).

Prominent structure-oriented notations include molecular interaction maps (MIMs) [36], process diagrams [33], and the Edinburgh [37] notation. To ensure efficient and correct use of these notations, many have been implemented in graph-based modeling tools (e.g., CellDesigner or Edinburgh Pathway Editor) and are accompanied by simulation environments (e.g. CellDesigner). MIMs however lack of software tools to assist the use of this graphical notation.

The graphical notation of the Narrator software tool emphasizes the development and exploration of *dynamic *models. This is explained by Narrator's main focus on the dynamic structure and properties of a biological system. With regard to SBML, structure-oriented notations correspond to SBML including Systems Biology Ontology terms for the SBML model elements, while dynamics-oriented notations can be understood as the notational counterpart of pure SBML, focusing on the dynamic description of biochemical models. In SBML level 2 version 2, Systems Biology Ontology (SBO) terms [41] can be optionally integrated into SBML model elements. The motivation of the SBO effort is to provide controlled vocabularies tailored to systems biology. In addition to facilitating consistency and sharing, SBO will also support visual rendering of the biochemical reactions.

A recently initiated effort called Systems Biology Graphical Notation aims to develop and standardize a structure-oriented graphical notation for representing biochemical networks [39]. Once available, this standard is likely to make user interfaces, model development, sharing and communication, and system interoperation more effective and efficient [39].

There are many methods listed in Table 1, such as methods for the automatic estimation of parameters values or steady-state analysis, that Narrator currently does not support but which do other modeling tools support. Copasi for instance integrates diverse numerical methods used in systems biology comprising deterministic, stochastic and hybrid numerical integration methods. Theses methods can, for example, divide networks into deterministic and stochastic subnetworks which run significantly faster than pure stochastic networks, or can deal with the numerical integration of deterministic networks in the presence of stiffness [34]. Similarly, Cellware provides an extensive algorithm library also comprising deterministic, stochastic and hypbrid integration methods [51]. The very special feature of Cellware however is that it is grid enabled supporting the distributed simulation of larger biochemical networks [12]. For 2D and 3D simulations Virtual Cell provides integrations methods for solving ODEs and partial differential equations. These methods can then be used for calculating the reaction and diffusion equation on arbitrary surfaces. Copasi, CellWare and Virtual Cell also provide parameter estimation and parameter scan components. Some tools such as JDesigner and CellDesigner have an interface to the Systems Biology Workbench (SBW) [50] which is an open source framework for connecting different software applications for modeling, analyzing or visualizing biochemical network models. Via the SBW interface, JDesigner for example uses the script based biochemical network simulation tool Jarnac [49] as a simulation server.

While ongoing developments implement more features such as bifurcation analysis, the objective of Narrator is not to cover the full spectrum of systems biology methods. Instead, the emphasis of Narrator and one of its major advantages over similar tools lies in the expressiveness and intuitiveness of its graphical notation and the sophisticated and highly usable implementation of a GUI supporting the use of this notation in the context of dynamics modeling and simulation. The focus of Copasi, for example, lies in the many numerical integration, parameter estimation and parameter scan methods. The graphical user interface of Copasi, however, is based on simple dialog boxes providing entry masks to define the chemical equation and rate expression for each reaction. The dynamics-based graphical design tools of JDesigner, Virtual Cell and CellWare, on the other hand, are based on directed hypergraphs or bipartite graphs. As shown in Figure 3 and discussed above (see section Co-dependence Notation), a possibility which is not explicitly allowed in such approaches is the ability to represent non-biological information sources such as temperature or osmotic pressure and their biological information processing accordingly. As also demonstrated above, the unique feature of Narrator, to link process nodes informationally, is a valuable modeling tool in many modeling scenarios and is not supported by the graphical notations of the abovementioned tools since edges in bipartite and hypergraph approaches can only relate biological species to processes or reactants to products. The fundamental structure of the structure-based graphical notation of CellDesigner is similar to directed hypergraphs and thus has the same restrictions in this respect.

## Conclusion

Narrator uses the simple, intuitive graphical notation of Co-dependence models [14] to express the dynamics of a biological system. A Co-dependence model describes dynamical relationships between the components of a system. Narrator simulates the behavior of such a system as an unfolding 'narrative' played out among the components.

Narrator is a stand-alone Java application for the graph-based modeling of dynamic network structures of biochemical systems. It extends the visual description of species transformation with the notion of information processing and therefore introduces a new concept into the multitude of graphical modeling tools used in the field of systems biology. Here, the provided intuitive design tool, which implements the Co-dependence Notation, supports the model building in a way that is accessible without an advanced mathematical background but which is still more oriented to mathematical dynamic systems than to biochemical pathway diagrams. This makes Narrator also applicable to any discipline requiring dynamic modeling.

The primary purpose of Narrator is to facilitate the creation and manipulation of computational models. Therefore, an ongoing effort in the development of Narrator is to maintain and improve the tool's SBML compatibility allowing a further exploration of Narrator models with simulation and analysis methods implemented in other tools.

## Availability and requirements

Narrator is available at [31] under the Lesser GNU Public license. The tool is entirely written in Java and runs with Java 5. It is platform-independent, which we have approved on Windows, Unix and Mac OS X machines. Additional information on installation and usage is also provided at [31]. The source code for Narrator is available at sourceforge .

## Authors' contributions

JJM and NMP developed the idea of Co-dependence modeling and developed the software tool Narrator. HF was involved in the implementation and gave a realistic user perspective. WD guided the development of this project. All authors read and approved the final version of the manuscript
